# Effectiveness of chlamydia Test and Treat strategy in preventing adverse pregnancy outcomes: protocol for a randomized controlled trial

**DOI:** 10.3389/fpubh.2023.1121888

**Published:** 2023-04-27

**Authors:** Lijun Liu, Changchang Li, Xuewan Sun, Bin Yang, Heping Zheng, Meng Li, Shujie Huang, Cheng Wang, Weiming Tang

**Affiliations:** ^1^Dermatology Hospital of Southern Medical University, Guangzhou, China; ^2^University of North Carolina Project-China, Guangzhou, China; ^3^Department of Obstetrics, The Seventh Affiliated Hospital of Southern Medical University, Foshan, China

**Keywords:** chlamydia trachomatis, pregnant women, testing, treatment, adverse pregnancy outcomes

## Abstract

**Introduction:**

Chlamydia trachomatis is one of the most common bacterial sexually transmitted infections worldwide, and is associated with an increased risk of adverse pregnancy outcomes. However, whether providing chlamydia screening and treatment during the first trimester of pregnancy could reduce adverse pregnancy outcomes is still not clear. This study reports a randomized controlled trial (RCT) protocol to evaluate the effectiveness of chlamydia Test and Treat during early pregnancy in preventing adverse pregnancy outcomes in China.

**Methods and analysis:**

This trial is a multi-center two-arm RCT targeting 7,500 pregnant women in early pregnancy (6–20 weeks). The inclusion criteria included: 18–39 years old, on their first antenatal visit, in the first trimester, and plan to deliver in the study cities. Following a block randomization procedure, every block of twenty women will be randomly assigned in a 1:1 ratio into two arms: (1) a Test and Treat arm in which women receive free chlamydia testing immediately after enrollment and people tested as chlamydia positive will receive standardized treatment and partner treatment; (2) a control arm in which women receive regular prenatal care without receiving testing during the pregnancy period, but collect urine samples and test them after delivery or indicating a chlamydia-related complication during pregnancy happens. The primary outcome is a composite of eight adverse events rate at delivery between two arms, including stillbirth, infant death, spontaneous abortion, preterm labor, low birth weight, premature rupture of membranes, postpartum endometritis, and ectopic pregnancy. Secondary outcomes include the cost-effectiveness of the intervention, the proportion of people tested with chlamydia infection, the proportion of tested-positive patients that received treatment, and the proportion of people who were cured 1 month after the treatment initiation. Urine specimens will be collected and tested for chlamydia by using Nucleic Acid Amplification Test. Data will be analyzed according to the intention-to-treat principle.

**Discussion:**

This trial will test the hypothesis that early testing and treating of chlamydia can reduce the risk for adverse pregnancy outcomes and may help in developing chlamydia screening guidelines in China and other countries with a similar prevalence of chlamydia infection.

**Trial registration:**

Chinese Clinical Trials Registry, ChiCTR2000031549. Registered on April 4, 2020.

## 1. Introduction

*Chlamydia trachomatis* is one of the most common bacterial sexually transmitted infections, with a prevalence of 3.2% among people 15–49 years old worldwide in 2020 (1). Meta-analyses have shown that chlamydia infection is associated with an increased risk of several pregnancies and fertility-related adverse outcomes (2). Yet, chlamydia infections are asymptomatic to a large extent and easily missed without screening, especially for women (3). Previous studies have explored the benefits of chlamydia screening and treatment during pregnancy in preventing adverse outcomes, and most of them support this intervention strategy (4–9). However, most studies took place in the US in the 1990s. Some interventions are still unavailable (for example, erythromycin is no longer recommended) due to gastrointestinal side effects (10) by then. The development of highly sensitive and specific nucleic acid amplification tests, more patient-friendly specimen collection methods, and highly effective treatment regimens (azithromycin and doxycycline) may make the screening more convenient and accurate and has the potential to further increase the effectiveness of the testing and treatment strategy, especially in countries with a high prevalence of chlamydia infection and adverse pregnancy outcomes, like China (prevalence of 4.7 to 10.2% in women) (11–13).

To prevent and control chlamydia, several countries have developed chlamydia screening policies, guidelines, or recommendations (14). For example, the US CDC recommends that women aged <25 years and those at high risk for chlamydia should be screened at the first prenatal visit and rescreened during the third trimester (10). Screening during early pregnancy aims to prevent adverse pregnancy outcomes (14). But prenatal chlamydia screening and treatment is not standard practice globally, and the effect size and cost-effectiveness data are scarce (2, 11), while only one study has been registered on ClinicalTrials.gov that offers STI screening and treatment to women at the first antenatal care service as an intervention (15). Presently, China is planning a comprehensive chlamydia prevention program. Still, before introducing such a program nationally, evidence from high-quality RCT is needed to show the effectiveness of screening compared with the harms at a reasonable cost (16). Therefore, we designed this trial to (1) evaluate the effectiveness of the chlamydia Test and Treat strategy in preventing adverse pregnancy outcomes; (2) assess the cost-effectiveness of the Test and Treat strategy.

## 2. Methods and analysis

### 2.1. Study design

This study is a multi-center two-arm RCT, with the main comparison being Test and Treat Strategy vs. the control group. Pregnant women will be randomly assigned in a 1:1 ratio into two arms: (1) a Test and Treat arm in which pregnant women in early pregnancy are offered free chlamydia testing, and the tested positive people will receive standardized treatment as well as treatment for partners; (2) a control arm in which pregnant women receive regular prenatal care without any intervention, but collect their urine for free chlamydia testing immediately either after delivery or indicating a chlamydia-related complication during pregnancy. Pregnant women will be recruited at three obstetrical departments in Foshan, Zhuhai, and Shenzhen of Guangdong Province, China. Every block of twenty participants in each of the two study sites will be randomly allocated into one of the two arms independently in sequential order.

**Figure 1:**
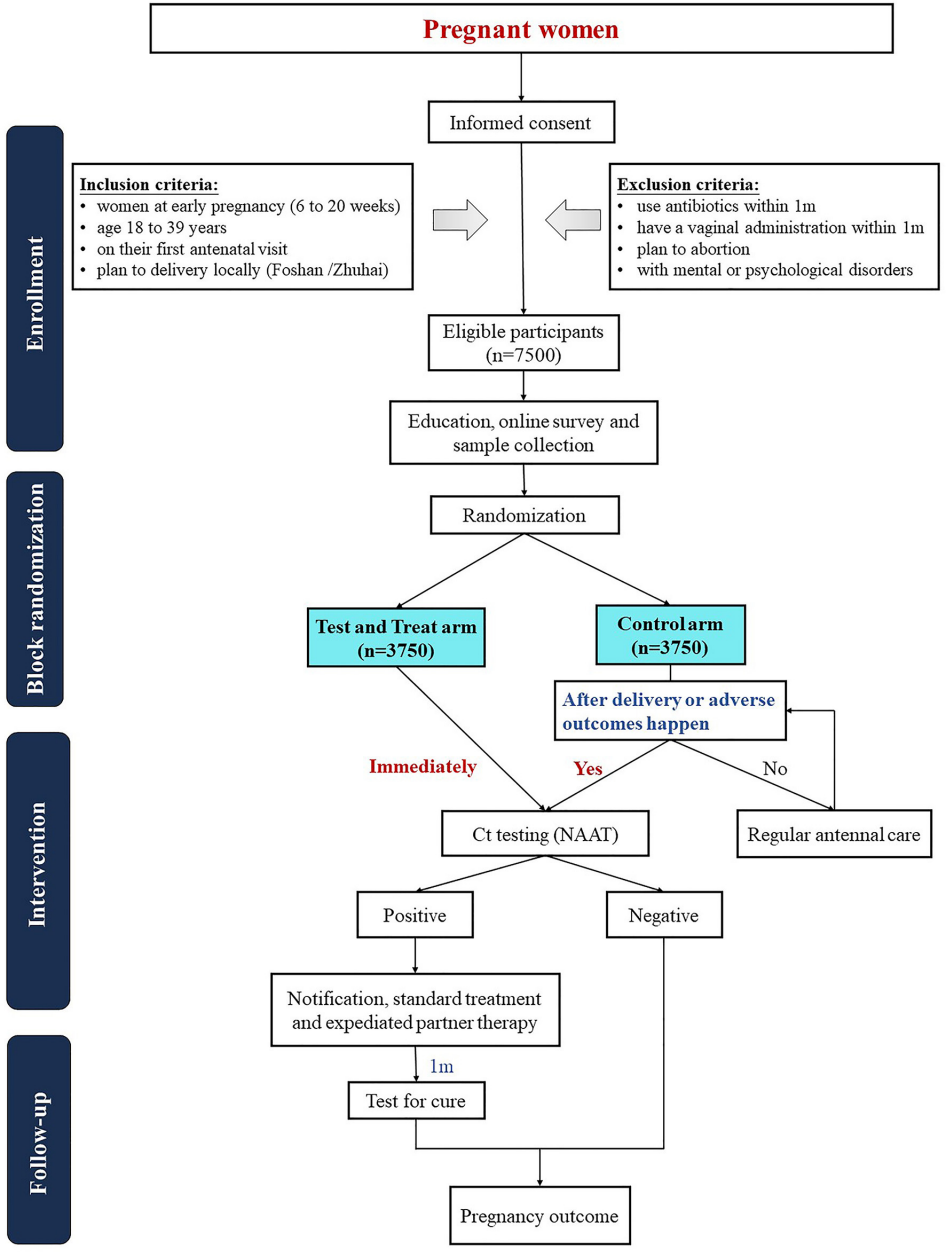
Flowchart.

We follow the SPIRIT guidelines, and a flowchart is shown in Figure 1.

### 2.2. Study settings and participants

This trial will be conducted in three local hospitals, one in each selected city. The three cities were chosen because they are well-developed and have diverse populations to guarantee representatives. The three hospitals are major medical institutions for maternal and perinatal health locally and have strong research teams. The professional staff at each site will be responsible for enrollment, health education, blood draws, results reporting, treatment and follow-up. Chlamydia testing will be confided to KingMed Diagnostics, a professional diagnostic company. All sites will follow the same study procedures.

The inclusion criteria are (1) women in early pregnancy (6 to 20 weeks); (2) age 18 to 39 years; (3) on their first antenatal visit; 4) plan to deliver locally. Exclusion criteria include (1) using antibiotics within 1 month; (2) having a vaginal administration within 1 month; (3) planning to have an abortion; (4) with mental or psychological disorders. All eligible participants must provide a mobile phone number for daily use in case positive results can be notified and further treatment can be assessed. Written informed consent will be collected from the participants after they are screened as eligible.

### 2.3. Randomization

At each site, participants will be assigned to either the Test and Treat arm or the control arm through a block randomization procedure with twenty women per block. The allocation sequence is determined by Stata 15.1 software (StataCorp, StataCorp LLC, College Station, Texas).

The study designer will randomly allocate all enrolled participants into two arms according to the sequence of visits and the allocation sequence mentioned above. After enrollment, urine samples will be sent to KingMed Diagnostics, a third-party independent testing agency that does not involve the study design. Then the study designer will tell the company which IDs should be tested immediately and which should be stored temporarily. Participants cannot determine their assignment, nor do staff in the field. Only researchers who design the study can access the group assignment. When the testing results from the participants in the intervention group are obtained, the study staff will notify the participants of the testing results and provide free standard treatment to the tested-positive participants as well as their partners.

### 2.4. Timeline

This study will span approximately 5 years, starting from May 2020. The first 2 months will include RCT preparations and a pilot study in Foshan (NCT03862495). RCT recruitment and implementation will be conducted in the following period until the pre-determined sample size is reached. The Dermatology Hospital of Southern Medical University will manage the study.

### 2.5. Intervention and control

#### 2.5.1. Test and Treat intervention

In China, pregnant women are recommended to register at the hospital early in pregnancy. At all sites, all eligible participants will receive information about chlamydia infection and its consequence by using a leaflet. After that, a brief study introduction will be delivered to each participant by the study staff in the local hospital. They will be told that if being allocated to Test and Treat group randomly, they will receive chlamydia testing immediately for free. If they are tested as chlamydia positive, free standard and partner treatment will be provided.

After informed consent elaborates on the study process, potential risks, benefits, and confidential clauses, participants will be asked to finish an online survey by scanning a QR code. The survey will collect information about socio-demographic characteristics, smoking and alcohol use, supplement intake (folic acid, Vitamin B12, etc.), quality of life, medical history (such as chronic diseases, STDs, and reproductive history), and sexual behaviors. Then, women will be guided to collect first-catch urine on their own, which will be stored at room temperature (2–30°C) overnight and then transported to KingMed Diagnostics for a chlamydia Nucleic Acid Amplification Test (NAAT).

Patients who test positive will be informed to come back to the hospital, usually within 1 week after enrollment, and provided a free standard treatment (Azithromycin 1 g orally in a single dose on the first day and 0.5 g on the second and third day, respectively) according to the latest Guidelines for the Clinical Diagnosis, Treatment, and Prevention of Sexually Transmitted Diseases (17) published by the National Center for STD Control, Chinese Center for Disease Control and Prevention. Sex partners are suggested to be managed by expedited partner therapy (10). Patients are suggested to inform their sexual partners and bring the same medications to them. To improve the treatment rate and adherence, the study staff will contact the tested-positive participants up to three times until they return to the hospital for treatment.

One month after treatment, they will be contacted again for a return testing of the cure by following the same testing strategy and evaluated about the adherence to treatment. Those who are still positive after testing for a cure will be treated with azithromycin again and required not to have sex within 1 month. Generally, all participants, regardless of their groups, will be followed up every 3 months to promptly identify adverse pregnancy outcomes or delivery.

#### 2.5.2. Control group

Pregnant women assigned to the control group will be encouraged to continue their usual antenatal care without extra interventions and accept the current guidelines, the WHO-endorsed “syndromic approach” (18). Procedures for informed consent, health education, online survey and sample collection will be identical to those of the Test and Treat group.

Chlamydia test will be conducted after delivery or in the event of adverse pregnancy outcomes, including stillbirth, infant death, spontaneous abortion, preterm labor, low birth weight (LBW), premature rupture of membranes (PROM), postpartum endometritis and ectopic pregnancy, which are suggested by our previous systematic review (2). Further treatment, follow-up, and sex partner management for patients with positive results will also align with those of the intervention group.

### 2.6. Laboratory testing

The urine samples should be transferred into cobas^®^PCR MEDIA tubes immediately after collection and then mixed upside down five times for further analysis, which will then be analyzed using Cobas^®^ 4800 CT DNA detection Kit (Roche Diagnostics, Basel, Switzerland) at KingMed Diagnostics company in Guangzhou, Guangdong Province.

### 2.7. Outcomes and measurements

#### 2.7.1. Primary outcomes

The primary outcome is to assess the impact of chlamydia screening and treatment of positive ones on eight adverse events rate et delivery in Chinese pregnant women. We'll compare the adverse events rate between two arms. The nominator will be the number of participants with at least one of the eight adverse events mentioned above in each arm. The denominator will be the total number of pregnancies in each arm. The definitions of those events are shown in Table 1, which refers to the International Classification of Diseases (19, 20).

**Table 1:** Definitions of primary outcomes based on the international classification of diseases (19, 20).

**Primary outcome**	**Definition**
Stillbirth	Refer to antepartum fetal death: a fetus that has suffered an intrauterine death after the 24^th^ week of gestation and before the onset of labor.
Infant death	Death between delivery and the first year of age (21).
Spontaneous abortion	A condition caused by immunological factors, abnormal ovum or uterine body, maternal disease or infection, or cervical incompetence. This condition is characterized by non-induced embryonic or fetal death or passage of products of conception prior to 22 weeks gestation or weighing less than 500 grams.
Preterm labor or delivery	A condition characterized by the onset of labor and delivery before 37 completed weeks.
Low birth weight of newborn	A pediatric condition in which the infant is born weighing between 1,500 and 2,499 g.
Premature rupture of membranes	Spontaneous rupture of fetal membranes before the onset of labor.
Acute endometritis [one of its most common manifestations is postpartum endometritis (22)]	A disease of the endometrium, caused by an infection with a bacterial or viral source. This condition is characterized by fever, lower abdominal pain, abnormal vaginal bleeding, or vaginal discharge. Confirmation is by a pelvic exam and identification of the bacteria or virus from a cervical swab, endometrial biopsy, or laparoscopy.
Ectopic pregnancy	Any condition characterized by implantation of the embryo outside the endometrium and endometrial cavity during pregnancy.

#### 2.7.2. Secondary outcomes

The major secondary outcome is the cost-effectiveness of intervention by comparing the two arms (incremental cost-effectiveness ratio, ICER). The other secondary outcomes include (1) the proportion of people tested with chlamydia infection, (2) the proportion of tested positive patients that received treatment, and (3) the proportion of people who were cured 1 month after the treatment initiation between two arms.

### 2.8. Sample size

The estimated sample size is 7,500 (3,750 for each arm). The sample size was calculated on the following: (1) incidence of a composite of adverse events of 16.2% in the control group based on a large survey in China, including spontaneous abortion, stillbirth, LBW, preterm birth, and very preterm birth (12); (2) proportional risk reduction of 17% (rate ratio = 0.83) based on the same survey mentioned above (taking LBW as a reference) (12); (3) 20% loss to follow-up; (4) significant level α = 0.05 (two-sided); (5) statistical power of 85%. Power analysis was implemented in the Power Analysis and Sample Size software 16.0 (NCSS, Kaysville, Utah, USA).

The possibility for an interim analysis after the enrollment of about 1,000 patients is included in the design, considering the large sample size needed and the possible adverse events.

### 2.9. Data analysis

#### 2.9.1. Primary analysis

Data will be analyzed according to the intention-to-treat principle. The difference in the incidence proportion of adverse pregnancy outcomes between the two groups will be compared using χ2 test. The primary analysis will evaluate the hypothesis that the Test and Treat strategy is effective in preventing adverse pregnancy outcomes. The general linear mixed model will be used to calculate the risk ratio, while potential confounders will be adjusted if the baseline characteristics of the participants are different between the intervention arm and control arm after the randomization.

#### 2.9.2. Secondary analysis

ICER will be used to determine the cost-effectiveness of screening pregnant women at early pregnancy for chlamydia compared with no screening. The cost includes a direct medical cost (testing, treatment, etc.), a direct non-medical cost (transportation, room, and board, etc.), and an indirect cost (time cost, training, staff salaries, etc.), assessed based on real-world charge, related survey and experts' advice.

#### 2.9.3. Subgroup analysis

Analysis of the effectiveness of early Test and Treat on outcomes by prespecified subgroups will be conducted, including (1) gestational week at enrollment (6–10, 11–15, 16–20 weeks); (2) whether positive patients finish standardized treatment (yes, no); (3) expedited partner therapy (yes, no); (4) age of the participants; (5) parity (primipara, multipara).

#### 2.9.4. Missing data plan

All pregnancy outcomes will be extracted from the Hospital Information System and Maternal and Child Health Information Platform as long as the delivery happens in the local city. Missing data cannot be avoided if they deliver a baby outside the range, and this part will be excluded or predicted if suitable. Besides, we set must-answer and jump terms in the online questionnaires, so missing data can be avoided to a large extent.

### 2.10. Quality control

Before the trial starts, we'll specify all procedures, including enrollment, sample collection, data management, handling of adverse events, etc. We will also organize training courses for all staff. All data generated, including questionnaires and general information, will be managed through Jinshuju, a professional data management platform (https://jinshuju.net/home). We will check data regularly in case of duplication (by examining ID numbers) and logic errors. All samples collected will be checked carefully to guarantee quality and integrity. Official reports of chlamydia testing will be issued by KingMed Diagnostic either on paper or on the website (https://kmos.kingmed.com.cn/km-ws/index).

### 2.11. Confidentiality

All data, including online-based questionnaires, testing results, and follow-up information, will be collected by designated staff and stored on secure professional online platforms (Jinshuju and KingMed Diagnostic website). Different accounts have different authorities. For example, the Zhuhai account cannot access the data collected from Foshan, and all information can be accessed with login information known only to the research team. After the completion of the trial, all electronic data will be downloaded from online platforms and managed with professional databases for at least 10 years, such as R and Stata, which can only be accessed with the permission of research team.

### 2.12. Monitoring

Suppose any person feels they are experiencing an adverse event, including the outcomes mentioned above, especially the person in the control group, or feels an unwanted effect. In that case, they will receive chlamydia testing immediately and receive related treatment or can withdraw at any time. The research team will provide participants with the phone number of staff and the Ethics Committee in case they have any questions.

## 3. Discussion

Chlamydia infection is one of the crucial risk factor for adverse pregnancy and fertility-related outcomes (2), and initiation of early screening and treatment of chlamydia infection among pregnant women has the potential to reduce the burden of adverse pregnancy outcomes (14). This two-arm trial aims to evaluate the effectiveness of the Test and Treat model intervention in preventing adverse pregnancy outcomes in China by using the most sensitive and specific testing, implementing the latest treatment guidelines, and assessing its cost-effectiveness. The study results will provide additional evidence and help in planning chlamydia screening programs in China and will guide the development of Chinese chlamydia testing guidelines among pregnant women.

Whether and when to test chlamydia among pregnant women is critically important but controversial. According to the up-to-date guidelines published by American Center for Disease Control and Prevention, chlamydia screening and treatment should be performed during the first prenatal visit to prevent adverse pregnancy events (14). With this in mind, limited prospective studies have confined their inclusion criteria to early pregnancy (4), so the association between treatment of chlamydia infection during pregnancy and improved outcomes may not be appropriate, and the evidence is of fair quality (23). Further results from this large RCT can be a good supplement.

When we initiate a new testing program, the cost is usually a big concern. Before the government decides to invest in chlamydia screening and treatment programs for pregnant women, which require substantial human, material, and financial resources, data on cost-effectiveness is necessary, especially for undeveloped areas (24). China is a representative of developing countries with a potentially high burden of chlamydia infection and a high prevalence of adverse birth outcomes (11). This trial can help further evaluate the cost-effectiveness of the Test and Treat strategy in China, which has the potential to extend to other resource-limited settings.

Our study also highlights some research priorities. There is an urgent need to design randomized studies in high-income countries. Notably, China (17) has a different treatment consideration (in terms of dosage) for chlamydia infection from that of the US CDC (10) and WHO (25). The effectiveness of Test and Treat intervention should be guided by local epidemiology and treatment guidelines. It is also important to differentiate the effectiveness in different age groups. Age is a substantial risk predictor for chlamydia infections (26), and currently, the age limitation of pregnant women that should be screened varies across countries (14).

Despite the public health significance of this exploration, some limitations need to be addressed. Women will be enrolled at early pregnancy and followed up until delivery, so a loss to follow-up is unavoidable, but we assume a 20% loss rate when calculating sample size, and we will contact them regularly to improve compliance. Besides, participants in the control group may take azithromycin and other antibiotics during pregnancy due to other diseases such as respiratory tract infections. This contamination may underestimate the effect size. To reduce this bias, we will collect all drug use records of participants from the Hospital Information System and Maternal and Child Health Information Platform. We will conduct a sensitivity analysis for women with chlamydia infection in the control group who take azithromycin.

Preventing and controlling chlamydia infection among pregnant women and improving reproductive health remains both a challenge and a global priority. This trial will provide additional important evidence from a resource-limited country. It also highlights the need for further research that considers local epidemiology and age limitations.

## 4. Trial status

At the time of finishing this protocol, participant recruitment is ongoing. This trial has been registered in the Chinese Clinical Trial Registry database (ChiCTR2000031549). Further modifications of protocol will be updated in this database, too. The RCT protocol conforms to the Standard Protocol Items: Recommendation for Interventional Trials (SPIRIT) 2013 statement.

## Ethics statement

The studies involving human participants were reviewed and approved by Institutional Review Board of the Seventh Affiliated Hospital of Southern Medical University (NHIRB [2020]1) and the Fifth Affiliated Hospital of Zunyi Medical University (CTIRB [2020]2). The patients/participants provided their written informed consent to participate in this study.

## Author contributions

WT, CW, BY, and HZ were responsible for conceptualization and methodology. LL, WT, and XS drafted the original paper. LL, CL, XS, SH, and ML were responsible for investigation, acquisition of data, and data curation. CW was responsible for funding acquisition. All authors contributed to the study conception and design and have read and approved the manuscript.
